# Urgent Care Centre Eligible Presentations in a Remote Emergency Department

**DOI:** 10.1111/1742-6723.70135

**Published:** 2025-09-09

**Authors:** Jack Johnstone, Chris Perry, Ellice Rigby, Lisa Capps, Richard Johnson

**Affiliations:** ^1^ Gloucestershire Hospitals NHS Foundation Trust Gloucester UK; ^2^ Alice Springs Hospital Alice Springs Australia; ^3^ Flinders Medical Centre Southern Adelaide Local Health Network Adelaide Australia; ^4^ NT Health Library Services Alice Springs Australia; ^5^ Australian Library and Information Association Canberra Australia; ^6^ Charles Darwin University Darwin Australia; ^7^ Flinders University Adelaide Australia

**Keywords:** emergency department overcrowding, healthcare access, low acuity presentations, Primary Health Care, Urgent Care Centre

## Abstract

**Objective:**

This study aimed to identify presentations to the Alice Springs Emergency Department that could be managed in an Urgent Care Centre (UCC).

**Methods:**

We reviewed 1 year of ED presentation data at Alice Springs Hospital (ASH) from August 2022 to August 2023 and used a sequence of exclusion criteria to identify patients most likely to be eligible for UCC management.

**Results:**

Our model indicated that 35.0% of ED presentations at ASH during this period could have been managed in a UCC. Only 41.5% of these presentations (14.5% of total presentations) occurred during UCC operating hours.

**Conclusions:**

According to this model, a significant proportion of ED presentations could potentially be managed in a UCC, although a large proportion of these occurred outside of UCC opening hours. The impact of the introduction of a UCC into a remote community on ED presentations, patient experience, patient outcomes and the broader system requires further study.

**Abbreviations**
ACEM: Australasian College for Emergency Medicine
ASH: Alice Springs Hospital
ATS: Australian Triage Score
BH: business hours
DNW: did not wait
ED: Emergency Department
GP: general practitioner
ICD‐10: International Classification of Diseases, 10th Revision
PHC: Primary Health Care
PPI: patient and public involvement
UCC: Urgent Care Centre
UCCEP: Urgent Care Centre Eligible Presentations

## 1 Introduction

### 1.1 Problem Description

Healthcare resources are finite, and it is essential to match services to the needs of the populations they serve. This is true in Mparntwe (also known as Alice Springs) in Central Australia, where healthcare delivery has challenges. There is a high prevalence of chronic diseases such as diabetes, end‐stage renal disease and rheumatic heart disease; there are complex social issues including alcohol misuse, domestic violence and housing instability; there is racism and the persistent effects of colonisation [1].

Alice Springs Hospital Emergency Department is an important part of the local healthcare system, providing accessible and free care for the community. Like many Emergency Departments, it is often used for low‐acuity presentations that could be safely and effectively managed elsewhere. It is frequently suggested that managing these low‐acuity presentations elsewhere could reduce pressure on Emergency Departments (ED) [2] as ED care is expensive, and ED overcrowding is associated with increased all‐cause mortality [3]. There is little agreement about how to redirect low‐acuity presentations safely and effectively. Decisions about where to seek healthcare ultimately rest with patients and are shaped by several factors, including alternative options, perceptions of urgency and past experiences [4]. The lack of robust definitions for ED presentations suitable for management outside the ED makes this more difficult [5].

An alternative to the ED currently being introduced across Australia is the Urgent Care Centre (UCC) [6], which offers a walk‐in service with extended hours, same‐day access to diagnostics and general practitioner‐led management, at a fraction of the cost of an ED [7]. One of these UCCs opened in Mparntwe in January 2024; however, there is little evidence whether a remote communities healthcare needs are well served by UCC care.

### 1.2 Available Knowledge

There have been multiple previous attempts to define ED presentations suitable for management outside the ED; however, they largely focus on the appropriateness for Primary Health Care (PHC). The difficulties reliably defining these presentations are evidenced by the wide range of estimates, which typically fall between 10% and 30% [8]. There is also some evidence suggesting higher rates of ‘GP‐type’ presentations in rural and remote areas, possibly due to limited access to PHC and after‐hours care [9]. There have been no previous attempts to quantify presentations appropriate for a UCC.

Part of the challenge is that the overlap between healthcare facilities has more dimensions than urgency alone. Attempts to identify presentations that might be managed outside of ED tend to rely on triage‐based measures of acuity such as the Australian Triage Score (ATS), which, while being a valuable indicator of urgency does not account for clinical complexity or correlate to the best care environment. An example is minor injuries, which could account for up to 37% of ‘GP‐type’ presentations [10, 11], but are both non‐urgent and well‐managed in ED, benefiting from same‐day radiology access and well‐defined referral pathways [12].

### 1.3 Rationale

The effective and efficient delivery of healthcare in remote Australia requires thoughtful resource allocation. Identifying and defining presentations better managed in a UCC will help to inform this. This information can inform triage protocols, guide service planning or strengthen communication between services. The challenges in Mparntwe may mirror those in other remote and rural communities across Australia, and information about ED presentations in Mparntwe might be relevant for other remote communities.

### 1.4 Specific Aims

- Identify ED presentations at ASH that would be appropriate for management in a UCC.
- Describe the demographic and diagnostic characteristics of these presentations.
- Establish a baseline against which future evaluations of the Mparntwe UCC's impact can be conducted.

## 2 Setting

Mparntwe is a remote community in Central Australia, 1500 km north of Adelaide and 1500 km south of Darwin, with a population of approximately 26,000 people. ASH is a 186‐bed hospital with a level 5 ED serving the local population plus approximately 25,000 additional people who live in remote communities across 1.5 million square kilometres. There are approximately 45,000 presentations to the ED per year. The hospital is a 5‐min walk from the town centre. There is some transport available for Aboriginal and/or Torres Strait Islander people provided by the Central Australian Aboriginal Congress (CAAC). PHC is provided by a combination of government‐run and Aboriginal Community Controlled remote and town‐based clinics and three private GP practices. The Mparntwe UCC, run by CAAC, opened in January 2024. It is located at the north end of the town, approximately a 20‐min walk from the hospital and town centre. It is open from 12:00 to 20:00 on weekdays, 12:00 to 16:00 on weekends and public holidays.

## 3 Methods

This study employed a retrospective observational design with expert clinician review to identify ED presentations that would have been suitable for management in a UCC.

### 3.1 Phase 1: Retrospective Audit of ED Presentations

A retrospective audit of routinely collected data was performed by the ASH clinical coding team. It included all patient episodes presenting to ASH ED between August 2022 and August 2023. The variables were triage category, mode of arrival, admission status, discharge diagnosis (modified ICD10 codes) and time of presentation.

### 3.2 Phase 2: Creation of a List of UCC‐Eligible Diagnoses

At discharge from ASH ED, presentations are coded using one of 1460 modified ICD10 diagnostic codes. Two ED clinicians (J.J. and E.R.) independently reviewed and classified all 1460 codes as either appropriate or inappropriate for management in a UCC, based on the Mparntwe UCC operational guidelines [13]. ED clinicians excluded a diagnosis code from UCC eligibility if they believed presentations with that diagnosis would be inappropriate for UCC management in > 90% of cases. This was a subjective judgement based on clinical experience. The final list of UCC eligible diagnoses was subsequently used as one of several exclusion criteria.

### 3.3 Phase 3: Application of Exclusion Criteria

The following exclusion criteria were applied to the dataset from Phase 1 to identify presentations not eligible for UCC management:

1. *ATS Category 1 or 2*—indicating the need for immediate or urgent resuscitation and treatment.
2. *Did not wait to be seen*—excluded from the primary analysis due to a lack of information about these presentations.
3. *Mode of arrival*—patients arriving by ambulance, police escort, or medical retrieval.
4. *Admitted to hospital*—implying the need for a level of care not available at a UCC.
5. *Diagnosis code*—presentations deemed inappropriate for UCC care based on clinician review.

### 3.4 Phase 4: Grouping of Presentations

Following exclusions, presentations were categorised into four groups:

1. All presentations: all ED presentations between 1 August 2022 and 1 August 2023.
2. UCC‐eligible: presentations that met all inclusion criteria for potential UCC management.
3. UCC‐ineligible: presentations that met one or more exclusion criteria.
4. UCC‐eligible during UCC business hours: presentations eligible for UCC management that occurred during Mparntwe UCC opening hours.

### 3.5 Data Analysis

Interrater agreement between the two clinician reviewers was assessed using Cohen's *κ* statistic. Exclusion criteria were applied to the dataset using Microsoft Excel. Descriptive statistics were used to summarise patient demographics, diagnostic categories and presentation patterns.

### 3.6 Did Not Wait to Be Seen

A fifth category, ‘Did not wait to be seen’, was created after the initial exclusion process. While excluded from the primary analysis, these cases were analysed separately, as they may provide valuable information for future research.

### 3.7 Diagnosis Code Mapping

The discharge diagnosis codes used in ASH ED differ from standard ICD10 codes but can be mapped to equivalent ICD10 categories. For reproducibility, analyses were based on the original ASH ED codes, except in Table 3, where the local code ‘Maltreatment syndrome, unspecified’ was replaced with its corresponding ICD10 code ‘Domestic assault’.

### 3.8 Ethics

Ethics approval was obtained from the Human Research Ethics Committee of NT Health and the Menzies School of Health Research (Reference number: 2023‐4743).

## 4 Results

### 4.1 Number of Presentations (Phase 1)

During the year between August 2022 and August 2023, there were 46,794 presentations to the ED in Mparntwe.

### 4.2 UCC‐Eligible Diagnoses (Phase 2)

The two clinicians agreed in 77.9% of cases on the first attempt at classification, with moderate agreement (*κ* = 0.527, *p* < 0.001). They subsequently came to a consensus for the remaining codes (Appendix S1).

### 4.3 Exclusions (Phase 3)

**FIGURE 1:**
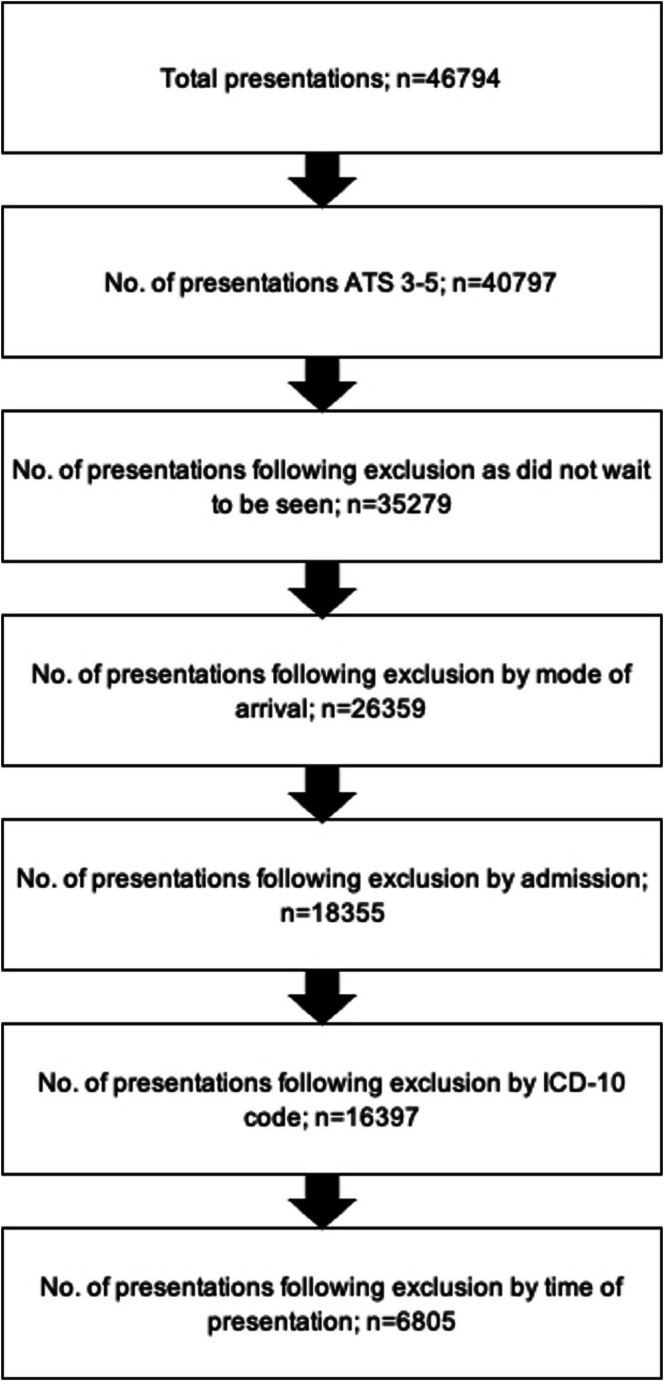
Stepwise exclusion of patients.

Table 1 shows the number of patients excluded by each separate exclusion criterion, broken down by triage category. Of the 46,794 presentations, 5535 did not wait to be seen, 13,030 were excluded by mode of arrival, 13,020 were excluded because they were admitted to hospital and 8742 were excluded based on the discharge diagnosis. Each exclusion is not mutually exclusive, with multiple exclusions often applying to the same presentation. Figure 1 shows a stepwise application of the exclusion criteria.

**TABLE 1:** Exclusions by triage category.

ATS	Total *N* (%)	Did not wait to be seen *N* (%)	Excluded on mode of transport *N* (%)	Excluded by admission *N* (%)	Excluded on ICD‐10 code *N* (%)	Excluded on time of presentation *N* (%)	ASH UCC eligible presentations *N* (%)
1	166 (0.35)	0 (0)	121 (0.93)	149 (0.8)	78 (0.89)	110 (0.38)	N/A
2	5831 (12.46)	17 (0.31)	3002 (23.06)	4444 (23.89)	2276 (26.04)	3691 (12.61)	N/A
3	17,786 (38.01)	660 (11.92)	7081 (54.39)	9985 (53.68)	4040 (46.21)	10,996 (37.57)	2046 (30.07)
4	20,781 (44.41)	4327 (78.18)	2769 (21.27)	3834 (20.61)	1733 (19.82)	12,870 (43.97)	4430 (65.1)
5	2230 (4.77)	531 (9.59)	47 (0.36)	188 (1.01)	615 (7.04)	1603 (5.48)	329 (4.83)
Total	46,794	5535	13,020	18,600	8742	29,270	6805

*Note:* ATS 1 and 2 patients are designated by N/A as they are excluded from the analysis.

### 4.4 Patient Groups (Phase 4)

There were 46,794 presentations in total. The UCC‐eligible group contained 16,397 (35.0%) presentations. The UCC‐ineligible group contained 30,397 (65.0%) presentations. 6805 of these cases (14.5% of total presentations) occurred during UCC operating hours.

### 4.5 Demographics

The primary readily available patient demographic data were gender, age and Aboriginal and/or Torres Strait Islander status. Table 2 shows that female patients presented more frequently than male patients (54.9% vs. 45.1%) with this difference reduced in the UCCEP group (52.0% vs. 48.0%). UCCEP tended to be younger than excluded patients. Aboriginal and/or Torres Strait Islander patients represented 47.6% of UCCEP, 65.5% of overall presentations and 75.1% of presentations excluded from management in a UCC.

**TABLE 2:** Demographics for each patient group.

	All presentations	UCC not eligible at any time	UCC eligible at any time	UCC eligible presenting during UCC business hours	Did not wait
Total number of presentations, *N* (%)	46,794 (100)	30,397 (64.96)	16,397 (35.04)	6805 (14.49)	5535 (11.83)
Indigenous status					
Aboriginal but not Torres Strait Islander *N* (%)	30,447 (65.07)	22,729 (74.77)	7718 (47.07)	3316 (48.73)	4403 (79.55)
Torres Strait Islander but not Aboriginal *N* (%)	49 (0.1)	24 (0.08)	25 (0.15)	16 (0.24)	3 (0.05)
Aboriginal and Torres Strait Islander *N* (%)	149 (0.32)	82 (0.27)	67 (0.41)	28 (0.41)	15 (0.27)
Not Aboriginal or Torres Strait Islander *N* (%)	16,046 (34.29)	7489 (24.64)	8557 (52.19)	3426 (50.35)	1109 (20.04)
Not stated *N* (%)	103 (0.22)	73 (0.24)	30 (0.18)	19 (0.28)	5 (0.09)
Sex					
Female *N* (%)	25,672 (54.86)	17,147 (56.41)	8525 (51.99)	3671 (53.95)	3099 (55.99)
Male *N* (%)	21,110 (45.11)	13,245 (43.57)	7865 (47.97)	3130 (46)	2436 (44.01)
Unknown/inadequately described *N* (%)	4 (0.01)	2 (0.01)	2 (0.01)	1 (0.01)	0 (0)
Other/indeterminate *N* (%)	8 (0.02)	3 (0.01)	5 (0.03)	3 (0.04)	0 (0)
Age					
Younger than 10 *N* (%)	6276 (13.41)	3044 (10.01)	3232 (19.71)	1422 (20.9)	627 (11.3)
10–19 *N* (%)	3970 (8.48)	2211 (7.27)	1759 (10.73)	819 (12.04)	509 (9.20)
20–29 *N* (%)	6983 (14.92)	4314 (14.19)	2669 (16.28)	1166 (17.13)	1025 (18.52)
30–39 *N* (%)	8574 (18.32)	5759 (18.95)	2815 (17.17)	1182 (17.37)	1219 (22.02)
40–49 *N* (%)	7675 (16.4)	5545 (18.24)	2130 (12.99)	805 (11.83)	993 (17.94)
50–59 *N* (%)	6857 (14.65)	4942 (16.26)	1915 (11.68)	686 (10.08)	763 (13.79)
60–69 *N* (%)	3983 (8.51)	2761 (9.08)	1222 (7.45)	459 (6.75)	282 (5.09)
70–79 *N* (%)	1726 (3.69)	1215 (4)	511 (3.12)	196 (2.88)	98 (1.77)
80–89 *N* (%)	654 (1.4)	525 (1.73)	129 (0.79)	62 (0.91)	17 (0.31)
90–99 *N* (%)	92 (0.2)	78 (0.26)	14 (0.09)	7 (0.1)	2 (0.04)
Older than 99 *N* (%)	4 (0.01)	3 (0.01)	1 (0.01)	1 (0.01)	0 (0)

*Note:* The percentages quoted in the first row are the percentage of total presentations to the ED. The percentages quoted in each column are a percentage of the total in a group for each category (indigenous status, sex, age).

### 4.6 Types of Presentation

The UCCEP differed from excluded presentations in terms of presentation types. Common UCCEPs included cases of acute viral illnesses, while excluded presentations were more likely to involve fluid overload (e.g., missed dialysis, a very common presentation in Central Australia), mental and behavioural disorders and chest pain. Table 3 shows the top 5 discharge diagnoses for each group.

**TABLE 3:** Top 5 discharge diagnoses.

	All cause, all time	UCC ineligible	UCC eligible	UCC eligible during UCC business hours
1	Mental and behavioural disorders due to use of alcohol including acute alcohol intoxication	Mental and behavioural disorders due to use of alcohol including acute alcohol intoxication	Acute upper respiratory infection, unspecified	Acute upper respiratory infection, unspecified
2	Chronic kidney disease, unspecified	Chronic kidney disease, unspecified	Viral infection, unspecified	Viral infection, unspecified
3	Fluid overload	Fluid overload	Other and unspecified abdominal pain	Other and unspecified abdominal pain
4	Domestic assault^a^	Domestic assault^a^	Mental and behavioural disorders due to use of alcohol including acute alcohol intoxication	Attention to surgical dressings and sutures
5	Other and unspecified abdominal pain	Chest pain unspecified	Attention to surgical dressings and sutures	Impetigo

^a^ Swapped from ‘maltreatment syndrome, unspecified’ which is the ICD‐10 mapped code from clinician coding ‘domestic assault’ on discharge from ASH ED.

### 4.7 Timing of Presentation

**FIGURE 2:**
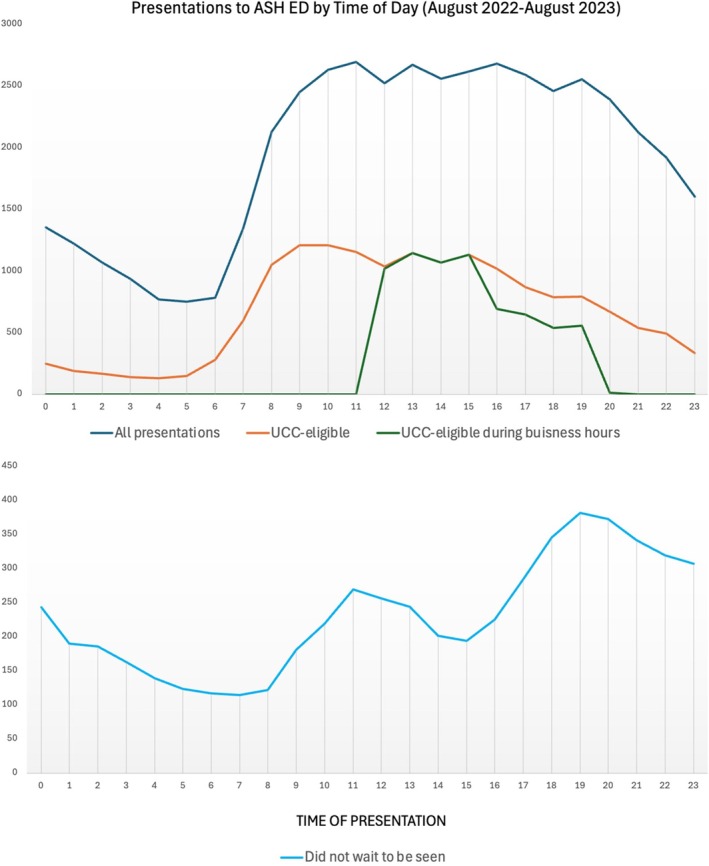
A graphical representation of ED presentations by time of day over a 12 months period, showing all presentations, those eligible for management in an urgent care centre at any time, those eligible who presented during UCC business hours and those who did not wait to be seen.

Figure 2 shows that UCCEP is present in a similar pattern to total ED presentations. There is a small morning peak outside of the current UCC business hours.

### 4.8 Did Not Wait

5535 (11.8%) of presentations did not wait to be seen by a doctor. Figure 2 shows the time of day that these patients present, with two peaks at 10 AM and 7 PM. 4421 (79.8%) of patients who did not wait to be seen identify as Aboriginal and/or Torres Strait Islander.

## 5 Discussion

### 5.1 Summary

This study is the first to systematically assess the proportion of ED presentations that could be managed in a UCC. A substantial share met criteria for UCC care, though many occurred outside current opening hours, limiting its potential as an alternative option.

### 5.2 Interpretation

Our findings are consistent with previous work showing that many low‐acuity ED presentations could be managed in alternative settings. The higher proportion observed here may reflect the broader scope of UCCs compared with PHC, or the lack of alternatives to ED in rural and remote areas. The types of presentations eligible for UCC care (acute viral illness) and the age (young adults) are consistent with expectations and the stated purpose of the UCC.

Of the large number of patients who left before being seen, 80% identified as Indigenous. This is a gap in the healthcare system that needs to be explored further, with a detailed investigation of who these patients are, why they did not wait and how their needs could be addressed. The implications for an UCC remain unclear; however, presentations by Indigenous patients were less likely to be eligible for UCC management. This could reflect the high burden of chronic disease and the complexity of health issues in this group but could also be influenced by the availability of free primary healthcare through the CAAC.

Most PHC‐appropriate presentations can be safely managed in a UCC; however, they may not be managed optimally. The strengths of PHC—continuity, coordination between services and chronic disease management—lie outside the scope of UCCs. While diverting low‐acuity presentations to UCCs may directly reduce their numbers in the ED, effective chronic disease management has been shown to reduce high‐acuity ED presentations, which would have a far greater impact on reducing ED overcrowding [14, 15].

The availability of appropriately trained healthcare professionals is a potential challenge that is particularly relevant for rural and remote communities. Difficulty recruiting in remote areas in Australia is well‐recognised. Recruiting from a finite pool of healthcare professionals is likely to exacerbate staffing pressures and require further expensive recruitment initiatives.

The model developed in this study could support ED triage and public education through the identification of patients suitable for UCC care. However, care‐seeking behaviour is shaped by complex factors [16]. While education and signposting are important, candidacy theory proposes that patients judge their own eligibility for different services. This means barriers to seeking care at alternative locations will be complex, particularly in a town where the reverberations of colonialism are still felt strongly. Past care experiences also influence future choices [17]. This suggests that a UCC may take time to establish. The quality and consistency of the care provided by the UCC will be important in determining whether it is used [17, 18]. The careful and ongoing assessment of patient experience will be essential in making the most of the new facility.

### 5.3 Limitations

The unique combination of health and social challenges in Mparntwe limits the generalisability of the findings. Other limitations include the use of retrospective data, reliance on ICD10 coding, exclusion of ‘did not wait’ patients and lack of patient and public involvement (PPI). The addition of a UCC clinician to the team would strengthen future studies.

## 6 Conclusion

A substantial proportion of ED presentations at Alice Springs Hospital could be managed in a UCC, though its potential as an alternative is limited by the current hours of operation. There are advantages to UCC care such as same‐day, free access for low‐acuity needs; however, they do not replace the continuity and coordination of PHC, and they are poorly equipped for effective management of chronic conditions. Care‐seeking behaviour is shaped by complex factors, and the success of a UCC will depend on delivering consistently high‐quality care and integrating seamlessly with other local services. Workforce shortages remain a persistent challenge in rural and remote settings, and these may be exacerbated by the new UCC. Future research should incorporate PPI, explore patient decision making qualitatively and capture patient experience. Direct assessment of UCC presentations and their effect on ED presentations will allow for the refinement of the model. Healthcare resources must ultimately be allocated in ways that recognise and respond to the needs of the communities they serve.

## Author Contributions

**Jack Johnstone:** data analysis, project design, writing (original draft, edits, resubmission), creation of figures. **Chris Perry:** data analysis, project design, writing (methods and edits). **Ellice Rigby:** writing – edits, expert opinion. **Lisa Capps:** literature review. **Richard Johnson:** conception, project design, data analysis, writing (edits).

## Ethics Statement

Approved by the Human Research Ethics Committee of NT Health and Menzies School of Health Research. Reference number 2023‐4743.

## Conflicts of Interest

Richard Johnson is currently the Deputy Director of Medical and Clinical Services at Alice Springs Hospital (ASH) and an Emergency Medicine Specialist at Alice Springs Hospital. At the time of conception of the project, Jack Johnstone was a Senior Resident Medical Officer in Alice Springs Hospital Emergency Department, and Ellice Rigby was an Emergency Registrar in Alice Springs Hospital Emergency Department.

## Supporting information


**Data S1:** emm70135‐sup‐0001‐DataS1.xlsx.

## Data Availability

The data that support the findings of this study are available on request from the corresponding author. The data are not publicly available due to privacy or ethical restrictions.
